# Social capital and its relationship to self-perceived health: National health survey in Colombia 2007

**Published:** 2014-03-30

**Authors:** Rafael de Jesús Tuesca-Molina, Eustorgio José Amed-Salazar

**Affiliations:** 1 Medical Surgeon Doctor of Preventive Medicine & Public Health. Professor in the Department of Public Health - Universidad del Norte. Barranquilla - Atlántico, Colombia. e-mail: rtuesca@uninorte.edu.co; 2 Nurse . Master´s in Public Health. Professor in the School of Nursing. Universidad de Sucre. Sincelejo. Sucre, Colombia. email: joseamed01@hotmail.com

**Keywords:** Cross-sectional, ethnicity, health status, socioeconomic factors, healthcare disparities, health perception

## Abstract

**Objective:**

To analyze the self-reported perceived health related to socio-demographic characteristics, social health inequalities and social capital in Colombia.

**Methods:**

This study is a cross-sectional design; data was obtained from the National Health Survey of Colombia 2007. Independent variables: socio-demographic characteristics; component variables: social health inequality and social capital. Dependent variable: self-reported health. Analysis of the relationship used logistic regression through OR and its confidence interval.

**Results:**

The determinant factors for a negative health perceptions are related to being a female (OR: 0.49 [0.47 to 0.52]), and in both genders being older than 37 years of age (OR: 0.72 [0.61 to 0.85]), living without a partner, black ethnicity, indigenous women (0.80 [0.69 to 0.94] and low economic incomes.

**Discussion:**

The relationship between social determinants and social capital in the perception of health shows inequities and indirectly reflects the level of health. Given the policies and the model of health, requires a rational adjustment of the goals, programs, and national and regional strategies with the object of improving the demand and quality of services.

## Abstract (es)

**Objetivo:**

Analizar la percepción de salud relacionada con características sociodemográficas, las desigualdades sociosanitarias y el capital social en Colombia.

**Métodos:**

Los datos provenientes de la Encuesta nacional de salud de Colombia 2007. Variables independientes: características sociodemográficas y variables componentes: desigualdad sociosanitaria y capital social. Variable dependiente percepción del estado de salud. Análisis de la relación mediante regresión logística a través de la OR y su intervalo de confianza.

**Resultados:**

Los factores determinantes para una percepción negativa de salud están relacionados con ser mujer (OR: 0.49 [0.47-0.52]), para hombres y mujeres ser mayor de 37 años (OR: 0.72 [0.61-0.85]), vivir sin pareja, ethnicidad negro/a; indígena en mujeres (0.80 [0.69-0.94] y bajos ingresos económicos.

**Discusión:**

La relación entre los determinantes sociales y el capital social en la percepción de salud muestra inequidades y reflejan indirectamente el nivel de salud; dadas las políticas y el modelo de salud se requiere un ajuste racional de las metas y programas y estrategias nacionales y regionales con el objeto de mejorar la demanda y la calidad de los servicios

## Introduction.

Self-perceived health refers to the self-assessment or consideration that an individual has of their own state of well-being, or state of clinical health 1 ^-^ 3. In spite of the controversy of this construct, the available literature assumes that its metric is widely used in national health surveys and that it can approximately measure the quality of life 4 ^,^ 5. It is suggested that this variable predicts morbidity and mortality and estimates functional decline in older persons 6 ^-^ 8. As well, it allows for an evaluation of tracking population groups with specific health problems, and is used to measure the effectiveness of interventions and health policies.

On the one hand, from the decade of the 90s the relationship between social capital and self-perceived health status has been evaluated. It is thought that improved growth in the state of health 6 ^-^ 9, physical activity, and increased use of local public services decreases mortality from chronic diseases, such as vascular brain, cancer, obesity, and behavioral factors such as smoking, psychosocial stress and suicide.

On the other hand, the estimation of social capital and self-perceived health measured at the community level determines patterns of political participation, trust, and establishment of networks 10 and permits assessment of the establishment of policies directed toward actions and interventions that promote health. Note that this tool is useful for epidemiology management.

In the nation of Colombia there is little research on the effects of social capital on health, as is the case in the rest of Latin America 8 and hence the importance of studying the relationship between socio-demographic factors and perceived health. A recent Colombian study considered some of the most important determinants of health status; this was measured differently. The role of income, age, gender, educational level, physical exercise, the health system, the regions of the country and the location of households in either urban or rural areas was emphasized.

National health surveys provide direct and relevant information over aspects related to health and the health system available; however, until now exploratory information has not been published regarding Colombians on different aspects that relate to perceived health. Hence, the object of this work is to analyze the health perceptions of Colombians related to independent variables or indices of social capital, health inequalities and social personnel. Likewise, it will allow the evaluation of health policy in order to estimate metrics derived from health surveys to monitor the level of welfare in the context of the reformulation and the rearrangement of the general social security system in Colombia 1.

## Materials and Methods

### Design and sample 

A cross-sectional study was conducted. The data came from the National Health Survey of 2007, released by the Ministry of Social Protection for research purposes. This survey used a cluster sampling design, that was stratified and multi-staged. Forty-one thousand five hundred and forty-three (41,543) households were selected with 164,474 persons,between the ages from 6 to 65 years and from all departments (i.e. provinces) of the country. The database was purged and the variables of interest were merged from Module 1 and 2 for households. Initially, a total of 80,628 participants were obtained and, consequently, persons with ages between 16 and 65 years were selected (population of an economically active age - PEEA) and the social capital instrument was administered to this subgroup. In the end there were 45,520 subjects.

This study was approved by the Research Ethics Committee of the University of North. This research was classified as without risks, according to Resolution 008430 of 1993 the Ministry of Health (Article 11), since the nature of this work analyzed data collected that were retrospective and from a secondary source.

### Procedures

In order to present the dependent variable (perceived health) dichotomously by individuals who responded to the survey question: "In general, how has your health been _____ (in the last thirty days? Options: Very good, Good, Fair, Poor and Very poor "). For this study the presentation of this variable is grouped into two dimensions: the first collapses the "very good" and "good" categories of health and calls this perception of health status "Acceptable" and the second group "Not acceptable", that collapses the categories of "Fair, Poor and Very Poor."

#### Principal component variables.

In order to present behavioral inequality within the variables, on the one hand and social capital, on the other, an analysis of the principal components in which all the questions, according to the questionnaire used in the household survey were applied, inquired concerning inequality and social capital. This analysis was performed because there were high correlations among the variables and this indicates that there was redundant information. Therefore, few factors, in this case two, explain much of the total variability. Therefore, for the factors of social inequality and social capital the distribution allowed sufficient members for each component variable.

The variables that were entered into the model for each component were evaluated according to the literature available for both contexts in order to obtain a synthesis of information and reduce the total number of independent variables. Each of the factors was given the same importance and components were extracted based on the largest percentage of variance explained in the grouping of the different variables of each component on the Cartesian plane.

#### Social inequality

Results from differences in living conditions, and from the environment in which an individual was born, grew-up, lives, works, ages and dies 11. The following variables were analyzed: educational level, type of work, membership in the health insurance system, socioeconomic strata, income and additional income for independent occupational activities, electric lighting, housing in a high risk area (prone to flooding, avalanche, mud slides, overflow of creeks and streams, geological faults).

#### **Social capital**. 

The various positions taken on the concept of social capital were reviewed; however, the nature of the variables assumed by the survey matches the model of Robert Putman, who represents the characteristics of social life translated into networks of civic associations, trust and norms. The following survey questions were analyzed: In which of the following groups did any member of this household engage in at least once a month: religious, athletic, political, cultural? In which of the following groups did any member of this household participate in at least once a month: community, entertainment, environmental, trade? Not counting household members or close relatives, does some person exist who would loan you a quantity of money, if needed? Are most people on this block /neighborhood willing to help when one of the neighbors has an emergency? Are you able to trust residents of this block/neighborhood? If a community project does not directly benefit you, but has benefits for many other people on the block/neighborhood, would you contribute time, money, other services? In the last year up until the present, have you or anyone in your household participated in any activity for the benefit of people on the block/neighborhood? If there were problems with the water supply on this block/neighborhood, what are the chances that people will cooperate to try to solve it?

The method of component analysis was used, a technique that reduces the number of variables involved in each factor and rediscovers groupings of variables in such a way that the variables in each group are highly correlated and can explain the majority of variability for each of factors.

**Figure 1.**
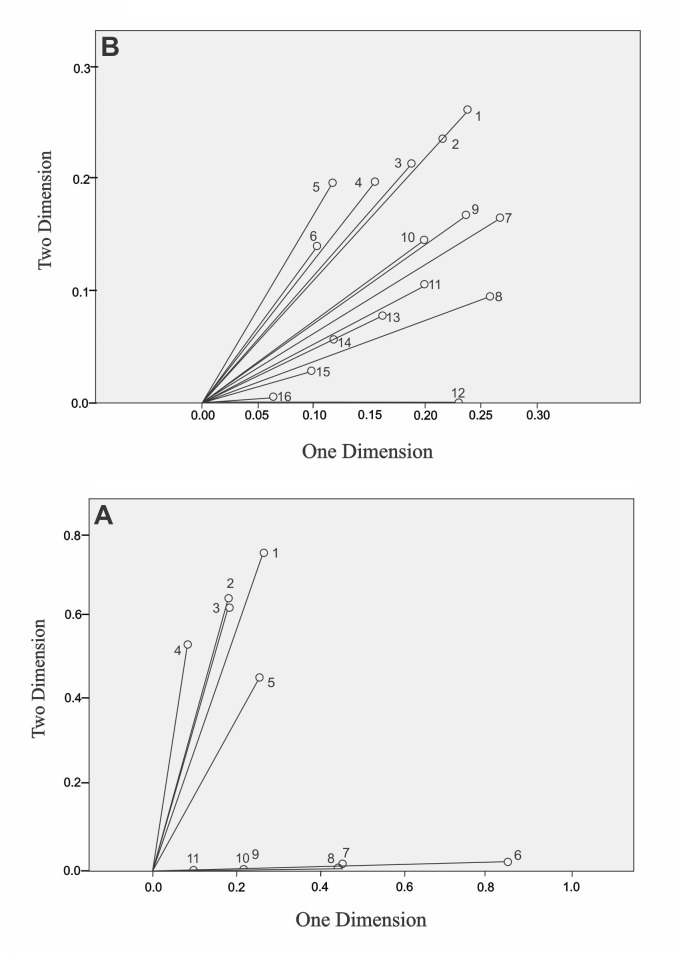
A. Descrimination variable component mesures inequality. B. Descrimination variable component mesures: Social capital.

Each resulting factor was scored on two dimensions and within each grouping of individual scores representative variables were selected for individual analysis and the component variables are as follows (Figs. 1A and 1B).

Model results for the first factor "Social-health inequality" show that Dimension 1 (Fig. 1A) is linked to the educational level variables, type of job, health system affiliation, socioeconomic status, income and additional income from independent occupational activities, and electric lighting. Given these relationships, the dimension can be called "Health system membership."

As for the second dimension (Fig. 1A), it is correlated with the variables: housing located in a high risk area (floods, avalanches, landslide/mudslide, overflows of creeks and streams, geological faults). Therefore, this component could be interpreted as "Housing in risk areas".

Figure 1B graphically accounts for such procedures taken to rename the dimensions of each factor.

The analysis of the second factor termed "social capital" results in two dimensions, where the variables with higher correlations were associated as follows:

The second dimension (Fig. 1B) is related to the variables: Are most people on this block/neighborhood willing to help when one of the neighbors has an emergency? If you had a problem with the water supply on this block/neighborhood, what are the chances that people will cooperate to try to solve it? Are you able to trust the residents of this block/neighborhood? If a community project does not directly benefit you, but has benefits for many other people on the block/neighborhood would you contribute time, money, other services? This dimension can be called "Contribution to the community."

The first dimension (Fig. 1B) has the following associated variables: In which of the following groups does a member of this household participate at least once a month: religious, athletic, political, cultural? Does some member of this household participate at least once per month in the following groups: community, entertainment, environmental, trade? Other than from household members or close relatives is there someone who would loan you a quantity of money, if needed? In the last year up until the present, have you or anyone in your household participated in any activity for the benefit of people on your block /neighborhood? Thus, the dimension can be renamed "Participation in cultural groups."

The following were used as independent variables: area of ​​residence (local governmental seat, population center and rural dispersement), region, cohabiting couple, occupation, reports belonging to ethnic group (indigenous, Afro/black descendants, gypsy and other) and level of education, age in decadal groups, region (Atlantic, Eastern, Central, Pacific and Bogotá), summary indices or component variables for inequality and social capital. 

### Analysis of data 

Analyses are presented separately for men and women. To estimate the distribution of the component variables, the distribution on the Cartesian plane and scores on matrix components were taken into account. The total percentage of the variance explained over 15% (Tables 1 and 2) was considered important in defining the number of components. A descriptive analysis was conducted, after which a chi square test was utilized for bivariate analysis with qualitative variables and the Student t-test was used for quantitative variables with a significance level of 5%.

**Table 1.**
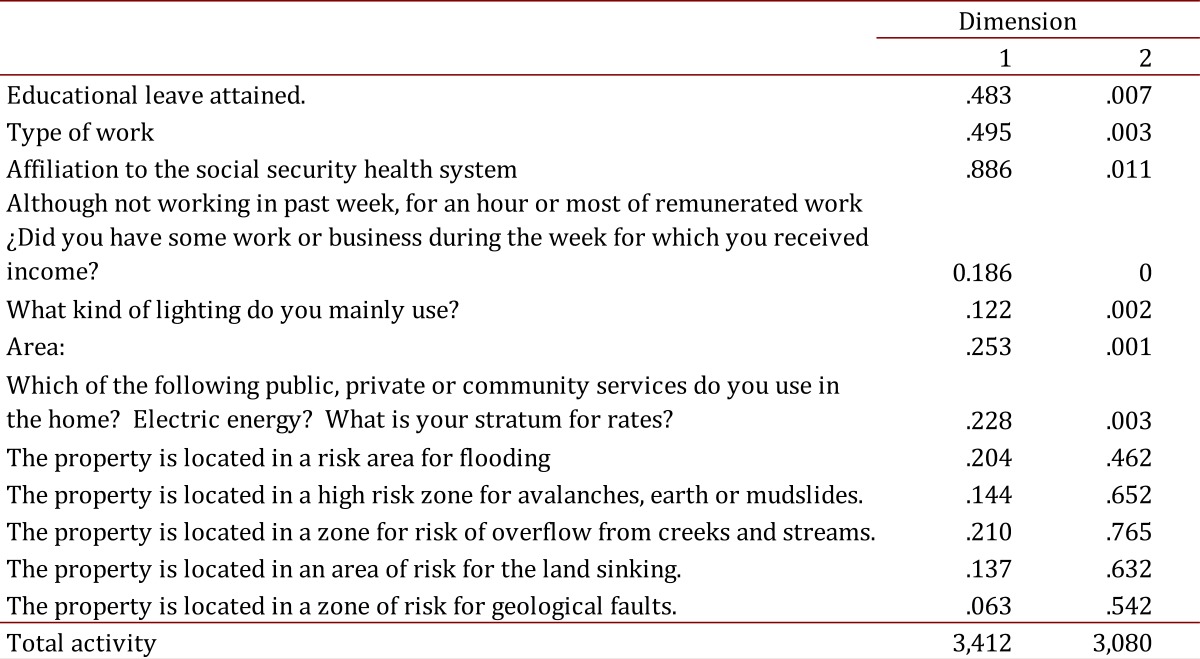
Component measures of the discrimination variable of inequality (coefficient matrix)

**Table 2.**
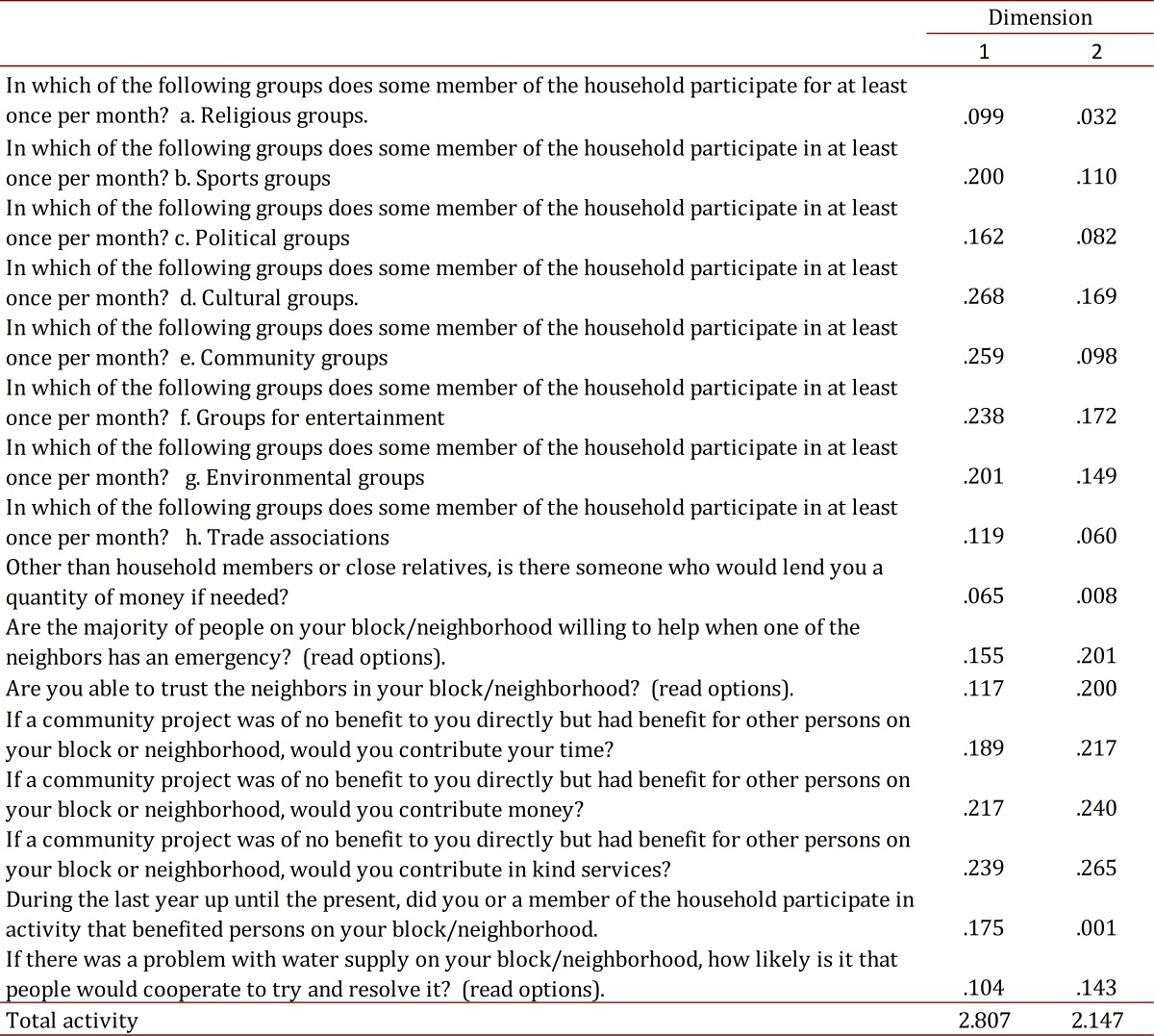
Variable measures of the discrimination component for social capital (coefficient matrix)

For the presentation of results of the analysis of multiple binary logistical regressions are shown for men and women with their odds ratios along with respective confidence intervals of 95%. The variables that were introduced to this analysis corresponded to those that in the bivariate analysis reached a p <0.20 with the SPSS version 19 statistical package being used. In the interpretation of the odds ratio values ​​above unity are expressed as "acceptable" or positive health perceptions and those below unity without being included were interpreted as "not acceptable" or negative perception.

## Results

Males reported having a higher proportion of acceptable or positive perceptions of health (76.28% versus 63.44%, p= 0.000) than women.

For women, unacceptable health perceptions are related to age from the age of 38, to living without a partner, to those who work in offices or labor activities independently, who act as a supervisor or employer, who report being Afro descendants and in those component variables of the second inequality index and second social capital index. On the other hand, the positive or acceptable health perception is related to levels of schooling at or above secondary school levels, women from palenques (rural communities formed by escaped slaves) and the dimension or inequality index one (Table 3).

**Table 3.**
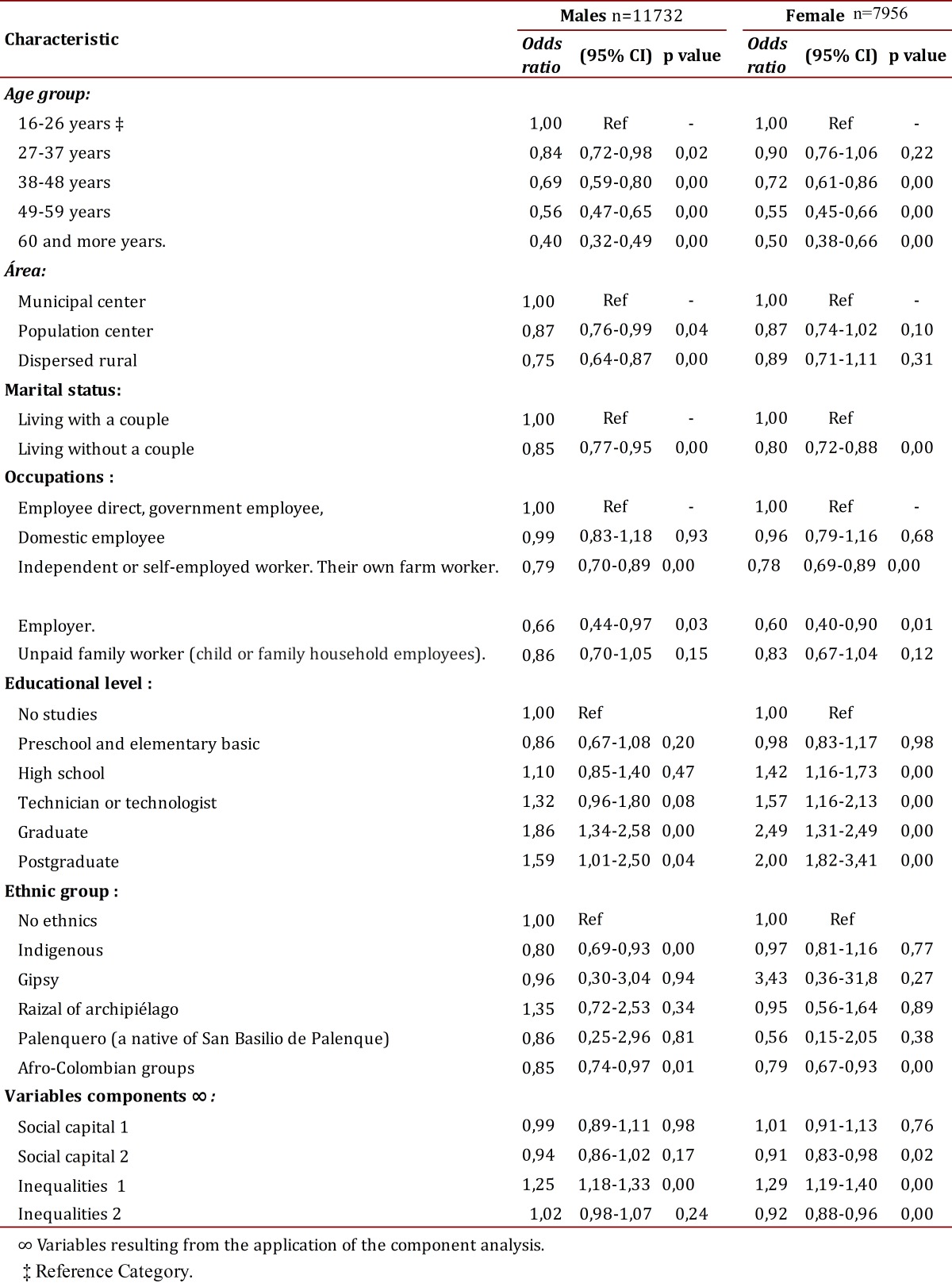
Multiple analyses of characteristics associated with self-rated health by sex

In men, unacceptable perceived health is related to age, from age 28 and upward the negative perception increases. In this unacceptable perception of health are added those living in villages and scattered rural areas, those living without a partner, independent professionals, supervisors or employers, the indigenous, Afro-descendants and those who do not contribute to their community. Men who report acceptable health perception are professionals with university and post-graduate degrees and the first inequality index (see Table 3).

## Discussion

Our results show social determinants of health of the proximal type, similar to other findings described that are related to this theme 3 ^-^ 5. Females perceive differences in self-perceived health compared to males; these findings suggest that arise in countries in transition, given that females experience greater life expectancy and greater co-morbitity 5 ^,^ 7 ^,^ 9 ^-^ 12. The findings related to age show that as one ages the levels of perceived health worsen, and this relationship is accentuated in women; most likely related to aging as it affects functionality, produces greater disability and the more likely development of depression 12.

In the field of health, it has been observed that those countries, cities and communities, in general, with greater levels of social capital tend to have a longer life expectancy and lower overall global morbidity and mortality and for specific causes 3 ^-^ 8, regardless of educational and socioeconomic levels. This association was also present in Latin America, although with some contextual differences; however, when the analysis was done by region the differences were not observed 8 ^,^ 10 ^,^ 12.

Regarding socioeconomic status assessed in terms of educational level, area of ​​residence and location in a high risk area, it showed that those who had a lower level of education and lived under vulnerable conditions reported negatively perceived health (not acceptable). These findings are similar to those of Humphries and van Doorslaer 13 ^-^ 15, and is confirmed with reports from some authors 5 who believe that increasing educational levels are associated with a lower probability of reporting poor health. This is very probably related to participation and appropriate use of preventive measures and those of health promotion 13 ^,^ 15 ^,^ 16. On the other hand, in highly unequal and inequitable societies, such as ours, in which the state is not visible in all parts of the territory, the community plays an important role because of the barriers to effective policy actions and state health and development programs 17.

Other studies have considered the effect of ethnicity on health status and found that ethnic minorities 2 are more likely to have a poor perception of their health status. These findings may relate to the fact of living in isolated areas that are distant from capital cities and accessibility to equitable health plans. Hence, it could be inferred that accordingly the black population of Colombia perceives a worse health status and has lower capital social capital 7 ^,^ 14.

Social inequalities and health (referred to as socio-health inequalities) were analyzed as a unified concept because of their mutual involvement and direct impact on the welfare of the population. These inequalities affect individuals in low income countries that implemented the neoliberal model. Women and ethnic groups are the least fortunate social classes 5 ^,^ 8 ^,^ 11. It is established that in states with neoliberal ideology models the ability to achieve autonomy on the part of the community generate uneven empowerment and unequal social capital than those with a supportive ideology of empowerment. Empowerment is constructed from new opportunity structures that seek to move towards a welfare state probably because it seeks to develop a redistribution of capital and opportunities that promote horizontal actions between the community and the state 15.

With regard to social capital, the available literature 19 ^,^ 20 ^-^ 22 presents various approaches and conceptualizations in their estimations; however, despite these variations, the common and relevant elements in the definition of social capital focus on estimating social participation being part of social networks and displaying mechanisms for cooperation, trust and norms. Therefore, Robert Putman proposes a cognitive and structural component. In this respect, networking norms and trust do not exist by themselves, as complementarity is required to address the problem of corruption and excessive bureaucracy.

The relationship between social capital and the state of health is given dimension in a direct and positive manner, it is hoped that with better social capital arising from social, educational, economic, network establishment, trust and supportive conditions, the perception of health will improve 19 ^,^ 21 ^,^ 22. It should be noted that in those countries in transition, social policies should aim at improving the social capital and the level of participation in a macro-contextual manner with the object of deploying policies that ensure inclusion and the state of well-being 15 ^,^ 22.

Despite the limitations of this study from being cross-sectional, it does not allow for establishing causality; however, but the fact that it employed an analytic strategy with logistic regression will raise awareness related to determinants of health perception and the fact of combining independent variables approximates relating the ecosystemic model together with social determinants of health. Perceived health is considered an essential element for determining priorities and planning health services and thereby guiding the actions of health promotion. The orientation of primary care and its interventions will be achieved through social and economic activities in the current Ten-Year Plan 2012-2021 by realizing substantial improvements in offerings and the quality of health services and social programs 22 ^-^ 23. 

The findings presented by Regidor *et al* 23 and Urbanos 24 show evidence of a reduction in health inequalities by promoting an increase in per capita income by region in Spain, also it identified increased educational levels as favoring better economic development and greater social justice. Furthermore, from these arguments the fact of including the measurement of inequalities and relating them to health status, as well as to social capital, can elucidate factors related to perceived health that may guide policy and actions health planning.

In our study, assessing the level of size of municipalities and geographical areas did not yield significant differences; however, these regions were introduced in the models by gender with the object of evaluating their effect. It presumably affirms that the second inequality component reflects the group weight of social determinants and from there the role of the region and area of residence can be argued while in the overall analysis inequalities by region are balanced, if regions are compared internally and globally and therefore are consistent with the findings of Rostila *et al* 25. 

On the other hand, the estimation of perceived health as one of the key elements in national or regional health surveys will permit guidance in health planning with policies that advance progress towards equity and solidarity for health programs and health advocacy to regions, to ethnic and other minority groups or those invisible to make apparent the beginnings of primary health care and intra-sectoral cooperation. In turn, this measurement is easy, simple and can be a marker for mortality and quality of health programs and social management.

The determinants of social capital in the positive dimension are closely related to the support network and this relation could likely be mediated by social support. Therefore, persons with more resources and social networks will have a better perception of their health, despite the fact that the role is not clear between them in the available research. This fact would facilitate progress in greater knowledge of social networking and the positive perception of health. The determinants identified in this research can make significant contributions for independently evaluating men and women, the effect of marital status (co-habitating), occupation, belonging to or identifying with ethnic groups, educational levels and social participation. Therefore, it would be expected that these determinants would be reflected in the Ten Year Plan for Public Health, 2012-2021, by rethinking the vision of public health in Colombia and placing the pursuit of equity and grounding the reorganization of health services with an inter-sectoral strategy of primary health care and homogeneity in the benefit plan for health.
